# VOLUNTEERING AMONG OLDER ADULTS AND EFFECTS OF ETHNIC MINORITY IDENTITY BEFORE AND DURING COVID-19

**DOI:** 10.1093/geroni/igae098.2139

**Published:** 2024-12-31

**Authors:** Eireann O’Dea, Andrew Wister, Lun Li, Sarah Canham, Barbara Mitchell

**Affiliations:** Simon Fraser University, Vancouver, British Columbia, Canada; Simon Fraser University, Vancouver, British Columbia, Canada; MacEwan University, Edmonton, Alberta, Canada; University of Utah, Salt Lake City, Utah, United States; Simon Fraser University, Vancouver, British Columbia, Canada

## Abstract

The ongoing COVID-19 pandemic has presented numerous challenges to older adults in Canada, including the ability to engage in volunteer work. This situation has created a new volunteer landscape in which common predictors of volunteering may have shifted. The purpose of this study is to improve understanding of the current social context surrounding volunteering in Canada, by a) determining changes in the associations between human, social, and cultural capital variables and volunteering among older adults and b) examining the potential relationship between ethnic minority background and volunteering among older adults, using data from the Canadian Longitudinal Study on Aging (CLSA), collected prior to and during the pandemic. This study utilized data from 24,306 participants (aged 55+) who participated in the CLSA Baseline, Follow-up 1 and the COVID-19 Study Baseline surveys. Results confirm a decrease in volunteering among CLSA participants during the early stages of the pandemic. When compared to pre-pandemic associations, volunteers during the early stages of the pandemic were more likely to be young-old (55-64), male, employed, and not involved in religious activities. Findings provide evidence of pandemic effects on volunteering among older adults in Canada.

